# Borrelial Diseases Across Eurasia

**DOI:** 10.3390/biology14101357

**Published:** 2025-10-03

**Authors:** Serena Bergamo, Giusto Trevisan, Maurizio Ruscio, Serena Bonin

**Affiliations:** 1 Dermatology Unit, ULSS 2 Marca Trevigiana, Ca’ Foncello Hospital, 31100 Treviso, Italy; serena.bergamo@aulss2.veneto.it; 2 Department of Medical Sciences, University of Trieste, 34149 Trieste, Italy; sbonin@units.it; 3 Clinical Pathology Unit, ASU GI-Azienda Sanitaria Universitaria Giuliano Isontina, 34128 Trieste, Italy; maurizio.ruscio@asugi.sanita.fvg.it

**Keywords:** *Borrelia*, Lyme, relapsing fever, Eurasia, ticks

## Abstract

**Simple Summary:**

*Borrelia* infections, caused by bacteria of the genus Borrelia, are transmitted by ticks and lice and affect human and animals across Eurasia. This review outlines the geographic distribution and ecological dynamics of Lyme borreliosis and relapsing fever, emphasizing the role of tick species, wildlife reservoirs, and environmental factors. It highlights how climate change and human activity are expanding tick habitats, increasing disease risk in new regions. The emergence of novel Borrelia species and the complexity of transmission cycles underscore the need of improved surveillance and diagnostic tools. Understanding these patterns is essential for developing effective public health strategies and anticipating future outbreaks.

**Abstract:**

This comprehensive review explores the distribution, diversity, and epidemiology of tick-borne borrelioses across Eurasia, focusing on Lyme borreliosis (LB) and other Borrelia-related infections. The genus *Borrelia* is categorized into three major groups, the Lyme Group (LG), the Relapsing Fever Group (RFG), and the Echidna–Reptile Group (REPG), each with distinct vectors, reservoirs, and pathogenic profiles. LB, caused by *Borrelia burgdorferi* sensu lato, is highly endemic in Europe and is increasingly reported in Asia, although it is underdiagnosed in Southeast Asia due to limited surveillance. This review details the ecological dynamics of tick vectors—primarily *Ixodes* spp.—and their vertebrate hosts, emphasizing the role of migratory birds and climate change in disease spread. It also highlights the presence of relapsing fever *Borrelia* species transmitted by soft ticks (*Ornithodoros* spp.) and the emergence of novel species such as *Borrelia miyamotoi* (RFG) and *Borrelia turcica* (REPG). This study underscores the need for harmonized surveillance systems, improved diagnostic tools, and integrated public health strategies to address the growing threat of borreliosis in Eurasia.

## 1. Introduction

*Borreliae*, members of the *Spirochaetaceae* family, are characterized by their distinctive spiral morphology. Currently, molecular techniques represent the cornerstone of Borrelia identification, with sequence-based approaches gaining increasing relevance in microbial research [1].

The genus *Borrelia* encompasses a diverse group of spirochetes, broadly classified as three major phylogenetic clades: the *Borrelia* Lyme Group (LG), the *Borrelia* Echidna-Reptile Group (REPG), and the *Borrelia* Relapsing Fever Group (RFG) [2]. While most species fall within these categories, several remain unclassified due to difficulties in cultivation and limited genomic data [3]. Borreliosis is usually transmitted by hard and soft ticks, except for Louse-Borne Relapsing Fever (LBRF) caused by *Borrelia recurrentis*, transmitted by the human body louse (*Pediculus humanus corporis*), a vector largely absent from Eurasia since World War II, and in some cases by *B. theileri*, which has been detected in the head lice of African pygmies [4]. Hard ticks (family *Ixodidae*) exhibit relatively long feeding durations—often several days—compared to soft ticks (family *Argasidae*) and the more recently described *Nuttalliellidae* [5].

Climate change has significantly altered the ecology of ticks and the epidemiology of tick-borne diseases (TBDs) through several interconnected mechanisms. Rising temperatures have enabled ticks like *I. scapularis* (vector of LB) and *I. ricinus* to expand into higher latitudes and altitudes, leading to the emergence of TBDs in previously unaffected regions, such as parts of Canada and Northern Europe [6]. Warmer winters and earlier springs have prolonged the active season of ticks, increasing the duration of host-seeking behavior and the risk of disease transmission [7]. Climate conditions such as higher humidity and milder winters have improved tick survival and reproduction with denser tick populations and a higher likelihood of human–tick encounters [8]. Climate change also impacts the establishment of invasive tick species such as *Hyalomma marginatum* and *Haemaphysalis longicornis*, which can carry novel pathogens. These species are now being reported in areas where they were previously absent [8]. These findings underscore the complexity of Borrelia ecology and raise important questions about the future distribution of borrelioses.

### 1.1. LG

To date, 24 species have been identified within the LG, 9 of which are known to be pathogenic to humans. Clinically, *B. afzelii* is predominantly associated with dermatological manifestations, whereas *B. garinii* and *B. bavariensis* are linked to neurological involvement. Lyme arthritis is most commonly attributed to *B. burgdorferi* sensu stricto (Bbss) [9]. Other pathogenic LG species include *B. spielmanii*, *B. lusitaniae*, *B. valaisiana*, *B. bissettii*, and *B. mayonii*, the latter being notable for its association with spirochetemia [10].

A recent addition to this group is *B. maritima*, isolated from coastal California, expanding the previously recognized diversity [3]. The identification of LG *Borreliae* can be performed through whole-genome sequencing and/or multi-locus sequence typing (MLST), based on eight genes, which enables unique genotyping and improves phylogenetic analysis [11].

Diagnosis of Lyme borreliosis (LB) typically relies on serological detection of anti-*Borrelia* antibodies via immunofluorescence assay (IFA), enzyme-linked immunosorbent assay (ELISA), chemiluminescent immunoassay (CLIA), or Western Blot (WB). Direct detection methods include culture in Barbour–Stoenner–Kelly H (BSK-H) medium and polymerase chain reaction (PCR) analysis of DNA extracted from skin biopsies or body fluids such as blood, cerebrospinal fluid, or synovial fluid [9].

LB is endemic throughout the Northern Hemisphere, with a particularly high incidence in North America and Europe. Its distribution is tightly linked to the presence of hard ticks of the genus *Ixodes*, including *I. ricinus* in Europe and North Africa (also *I. inopinatus*), *I. persulcatus* in Asia and Eastern Europe, and *I. scapularis* and *I. pacificus* in North America. Conversely, LB is rarely reported in the Southern Hemisphere [3].

Ticks of the genus *Ixodes* acquire *Borrelia* from reservoirs of *Borrelia*-infected vertebrate hosts. The composition of local wildlife and the dynamics of tick–host interactions are critical to the persistence and spread of LB in natural ecosystems. *Ixodes* spp. ticks thrive in humid environments (>80% relative humidity) and can colonize a wide range of habitats, such as woodlands, grassy meadows, broad-leaved forests, and pastures. Their abundance is influenced by vegetation and host density, particularly domestic animals (e.g., sheep and cattle) and wild fauna.

Vertebrates play dual ecological roles:

- As tick hosts, wild species such as roe deer, red deer, foxes, and hares facilitate tick dispersal.
- As reservoir hosts, small mammals (e.g., rodents) and birds maintain and transmit *B. burgdorferi* sensu lato (Bbsl) in nature.

Migratory birds are particularly important in the long-distance dissemination of infected ticks, potentially bridging endemic and non-endemic regions, including intercontinental spread—although LB remains rare in the Southern Hemisphere [12]. In Southeast Asia, LB is likely underreported due to limited diagnostic infrastructure and surveillance [13].

### 1.2. REPG

The REPG was established following the identification of *B. turcica* in *Hyalomma aegyptium* ticks from tortoises in Türkiye [14]. This group currently includes *B. turcica* and *B. tachyglossi*, expanding the ecological and evolutionary scope of *Borrelia*.

### 1.3. RFG

Relapsing fever *Borrelia* species are also present in Eurasia, typically transmitted by soft ticks of the genus *Ornithodoros*. The epidemiological distinction between the vectors of LB and Tick-borne Relapsing fever (TBRF) is significant:

- LG *Borreliae* exhibit limited vertical transmission in *Ixodes* spp.
- RFG *Borreliae* are efficiently transmitted vertically in *Ornithodoros* spp., which may also act as reservoirs, as seen with *O. moubata* [15].

Some RFG species are also transmitted by hard ticks, a condition termed Hard Tick-Borne Relapsing Fever (HTBRF). *B. miyamotoi*, a notable HTBRF agent, is transmitted by *Ixodes* spp. (*I. ricinus*, *I. scapularis*, and *I. persulcatus*) [16], capable of vertical transmission [17], and has been detected in various wildlife reservoirs [18]. *B. theileri*, another HTBRF *Borrelia*, is transmitted by *Rhipicephalus* spp. and *Margaropus australis* and affects primarily livestock, but is not known to infect humans, although it causes significant economic losses in the agricultural sector [19,20]. *B. lonestari*, transmitted by the Lone Star tick (*Amblyomma americanum*), has been associated with human illness, but its pathogenic role remains uncertain [21].

## 2. Borreliosis in Eurasia

Europe lies entirely within the Northern Hemisphere, whereas parts of Asia—specifically Indonesia, East Timor, and Brunei— are located in the Southern Hemisphere. Additionally, the southernmost regions of Malaysia, Singapore, the Philippines, Myanmar, Thailand, Cambodia, and Vietnam—extend into the Southern Hemisphere. This geographical diversity contributes to varying ecological and disease dynamics across the continent.

This review provides an overview of the distribution of borreliosis in Europe, where Lyme borreliosis is highly endemic, followed by a more detailed analysis of its presence in Asia. The Asian region encompasses countries in both hemispheres and is characterized by limited and heterogeneous epidemiological data. Several areas may be considered transitional zones, situated between regions where LB is well documented and those where it is either non-endemic or remains unreported. In addition to LB, other *Borrelia*-associated diseases are discussed, including those caused by species within the RFG and the REPG.

Overall, several *Borrelia* species are pathogenic to both humans and other mammals, while others infect animals or are not pathogenic. Vector ecology plays a crucial role in the transmission dynamics of *Borrelia* infections. In Eurasia, human infections are primarily transmitted by hard ticks of the genus *Ixodes* and soft ticks of the genus *Ornithodoros*, as summarized in Table 1. Animal borreliosis (see Table 2) is vectored by hard ticks belonging to the genera *Ixodes*, *Rhipicephalus*, *Haemaphysalis*, *Hyalomma*, and *Amblyomma*. Soft ticks, mainly of the genus *Ornithodoros*, are responsible for transmitting Soft Tick-Borne Relapsing Fever (STBRF). Additionally, ticks of the genus *Argas* can transmit relapsing fever to birds, while *Carios* spp. are associated with transmission in bats.

Some species primarily infect animals, which has important implications for both public health and the economy. For instance, *B. theileri* and *B. persica* can cause disease and abortion in livestock, resulting in significant economic losses.

Other species, such as *B. hispanica*, *B. duttoni*, and members of the *B. burgdorferi* sensu lato complex, are capable of infecting domestic animals and may pose a zoonotic risk, potentially facilitating transmission to humans.

### 2.1. Europe

In the Northern Hemisphere, *Ixodes* spp. ticks are more abundant in cool, humid microclimates. Their activity peaks during spring and autumn in temperate regions, while high summer temperatures often drive them to adopt an endophilic (indoor or sheltered) lifestyle. Rainy climates with high humidity levels (90–95%) further facilitate their proliferation.

In Europe, the primary vector of LB is *I. ricinus*, whose distribution has expanded significantly in recent decades. This expansion is attributed to a combination of climatic, ecological, landscape, and anthropogenic factors [42]. *Ixodes ricinus* exhibits high ecological plasticity, enabling it to survive in suboptimal climatic conditions and colonize high-altitude environments, reaching elevations up to 1800 m above sea level. These adaptive traits, coupled with land-use changes and biotope transformation, influence tick habitat availability and increase the likelihood of host–tick–pathogen interactions, thereby elevating the risk of tick-borne pathogens transmission [43]. Moreover, *I. ricinus* exhibits low host specificity, parasitizing a broad range of vertebrates, including mammals (humans included) and birds [44].

**Figure 1:**
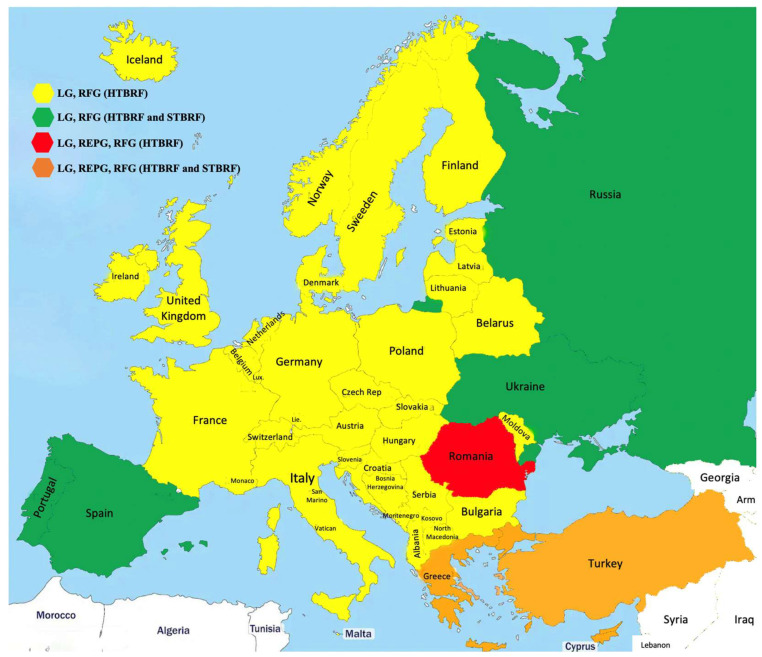
*Borreliae* distribution in Europe.

The distribution of tick-borne pathogens is primarily governed by tick population density and the presence of competent reservoir hosts. Figure 1 illustrates the distribution of *Borreliae* across Europe. In this region, primary reservoirs for Lyme Group *Borrelia* species include small- to medium-sized rodents, such as *Clethrionomys glareolus* [45], alongside various bird species, especially passerines like *Turdus merula* (the common blackbird) [33]. Transmission among ticks can also occur via co-feeding, a mechanism whereby multiple ticks feed in close proximity on the same host, facilitating pathogen exchange even in the absence of systemic infection [46].

Estimating the true incidence of Lyme borreliosis in Europe is challenging due to substantial heterogeneity in surveillance systems. These range from passive to mandatory reporting and from sentinel site monitoring to nationwide data collection. Variations in case definitions (clinical, laboratory-based, or both) and diagnostic methodologies further contribute to underreporting, particularly in countries where notification is not mandatory.

The average population-weighted incidence across Europe is estimated at 22 cases per 100,000 inhabitants per year [47], with at least 200,000 new cases annually [48]. Approximately 24% of the European population resides in regions classified as high-incidence areas. To improve the comparability and understanding of LB incidence across countries, standardized surveillance systems and harmonized case definitions are urgently needed [49].

Documented cases of LB have been reported in numerous European countries, including Austria, Belgium, Bosnia, Bulgaria, Croatia, the Czech Republic, Denmark, Estonia, Finland, France, Germany, Greece, Hungary, Iceland, Ireland, Italy, Norway, Poland, Russia, Slovakia, Sweden, and the United Kingdom [49].

In 2016, approximately 85,000 clinically manifest LB cases were reported across Europe, with the highest frequency observed in temperate regions. However, this figure likely underrepresents the true burden due to underreporting and variability in surveillance practices [49]. Notably, Iceland—previously considered a low-incidence country (2 cases per 100,000 inhabitants/year)—has experienced a significant rise in incidence, averaging a 21% increase over the past 12 years [50].

Population-based LB incidence is typically expressed as cases per 100,000 inhabitants per year. Regions with incidence rates exceeding 10 cases per 100,000 are considered high-risk. The highest national incidence rates have been reported in Estonia, Lithuania, Slovenia, and Switzerland, each exceeding 100 cases per 100,000 population per year. France and Poland report intermediate rates (40–80/100,000), followed by Finland and Latvia (20–40/100,000). The lowest incidence rates (<20/100,000) are observed in Belgium, Bulgaria, Croatia, England, Hungary, Ireland, Norway, Portugal, Romania, Russia, Scotland, and Serbia [49]. Nevertheless, epidemiological data should be evaluated with care because they do not always reflect the real situation. This is the case in Serbia, where reporting has not been obligatory since 2016. In Italy, LB is present nationwide, with a particularly endemic patterns in Alpine regions [51].

In European Russia, the southern forested regions have experienced both an extension of the tick activity season and an increase in LB incidence rates. The primary vector in these areas is *I. persulcatus*, capable of transmitting *B. garinii*, *B. afzelii*, and *B. bavariensis* [52]. In Europe, the following pathogenic *Borrelia* LG genospecies have been identified: *B. afzelii*, *B. garinii*, *B. bavarensis*, *B. burgdorferi* sensu stricto (Bbss), *B. lusitaniae*, *B. valaisiana*, *B. spielmani*. *B. garini* is most prevalent in Central Europe, whereas *B. afzelii* predominates in Northern and Southern Europe. Notably, *Fratercula arctica* birds (Atlantic puffins) have been reported to carry *I. uriae* ticks infected with *B. garinii* to the Faroe Islands [22].

Tick-borne relapsing fever borreliosis is also present in Europe, particularly in the form of Hard Tick-Borne Relapsing Fever, caused by *B. miyamotoi*. This species shares the same tick vectors as *B. burgdorferi* sensu lato, primarily *Ixodes* spp., and has been detected in nearly all European countries [53].

Additionally, several European countries report cases of Soft Tick-Borne Relapsing Fever. For example,

- *B. caucasica* is transmitted by *O. verrucosus* in Ukraine and parts of European Russia [54,55].
- *B. hispanica* is transmitted by *O. erraticus* in Spain, Portugal, and Greece [56]. This species can infect both humans and domestic animals, including dogs and cats, with cases reported in Córdoba, Valencia, and Seville [57].

Ticks carrying *Borrelia* species responsible for relapsing fever have been reported on all continents except Antarctica and Australia. While molecular diagnostic techniques (e.g., PCR and sequencing) are required for precise species identification, microscopy remains the gold standard in many clinical settings, particularly in resource-limited areas.

The hallmark clinical feature of tick-borne relapsing fever is its recurrent febrile episodes, which give the disease its name. These episodes are typically separated by afebrile intervals and are caused by waves of spirochetemia. A notable complication during treatment is the Jarisch–Herxheimer reaction, an acute inflammatory response that may occur shortly after the initiation of antibiotic therapy. It is characterized by fever, chills, hypotension, and worsening symptoms, and is thought to result from the rapid lysis of spirochetes and the release of endotoxin-like substances [58].

### 2.2. Asia

In Southeast Asia, a total of 97 tick species have been documented, including 42 species of *Haemaphysalis* and 14 species of *Ixodes*.

*Borrelia* species have been detected in both hard and soft ticks in the region. Potential reservoir hosts include rodents, birds, lizards, and snakes. Individuals engaged in agricultural activities near forested areas are at increased risk of tick exposure. Moreover, anthropogenic factors such as deforestation and urbanization can disrupt natural habitats, potentially expanding the geographic range of tick-borne and zoonotic diseases by increasing human–animal contact [59].

In Asian countries, the distribution of ticks and their associated pathogens is influenced by a combination of climatic, environmental, and ecological factors. Studies analyzing ticks collected in China, Japan, Malaysia, Mongolia, Pakistan, Asian Russia, South Korea, Thailand, and Turkey have confirmed the presence of *B. burgdorferi* sensu lato in ticks of the genera *Ixodes* and *Haemaphysalis* [60].

In northern Mongolia, China, and Japan, the predominant vector is *I. persulcatus*, which is capable of transmitting *B. afzelii* and *B. garinii* (Asian variant NT29), but not *B. burgdorferi* sensu stricto. The most frequently identified reservoir hosts include *Apodemus ainu* in Japan and *Niviventer confucianus* in China, Vietnam, Laos, and Cambodia [61].

In China and Thailand, *I. granulatus* and *Ixodes columnae* are important vectors, whereas in Japan and Korea, *Ixodes nipponensis* is responsible for the transmission of *B. valaisiana* (strain Am 501) [62].

Clinical cases of LB have also been reported in several Asian countries (see Table 3), including Korea, Nepal, China, and Japan. In Indonesia, Malaysia, and Singapore, LB has been identified in human serum, primarily through IgG and IgM ELISA testing. *Borrelia* of the Lyme group has been detected by PCR in ticks collected from host animals such as *Sundamys muelleri* and *Python* species in Malaysia, Thailand, and Laos. These findings confirm the presence of Bbsl infections in Southeast Asia, although the actual number of cases is likely underestimated [63].

Soft ticks of the genus *Ornithodoros*, vectors of STBRF, are also widespread in Asia, as reported in Table 4. *B. persica*, transmitted by *O. tholozani*, is prevalent in the region. Other STBRF-associated *Borrelia* species reported in Asia include *B. baltazardii*, *B. graingeri*, and *B. microti* [94].

**Figure 2:**
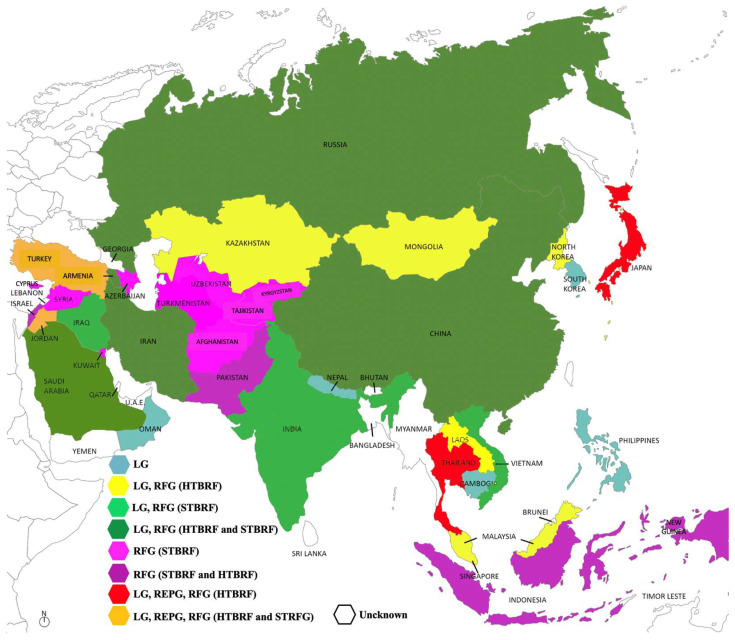
*Borreliae* distribution in Asia.

In some Asian countries, however, data remain scarce. No cases have been reported in the literature for Brunei and Vietnam [63]. In Bhutan [108] and Myanmar [109], a study aimed at identifying zoonotic pathogens in rodents and ticks yielded negative results for *Borrelia* spp. A summary of the distribution of different *Borrelia* groups across the region is provided in Figure 2 [63]. In the following sections, countries where *Borreliae* have been documented are listed in alphabetic order.

#### 2.2.1. Asian Russia

In Eastern Russia, *I. persulcatus* is the vector of LG *Borrelia* species, but not of Bbss. This pattern mirrors findings from wild rodent hosts such as *Clethrionomys rufocanus* and *Apodemus peninsulae*, and aligns with results from studies conducted on *I. persulcatus* and wild rodents in Hokkaido, Japan [110]. The tick *I. pavlovskyi* has been found to be exclusively infected by *B. garinii* [64].

In Eastern Russia, *B. afzelii*, *B. garinii*, *B. bavariensis* (LG), and *B. miyamotoi* (HTBRF) have been identified [111]. The *B. garinii* NT29 group, transmitted by *I. persulcatus*, is present in both Asian Russia and northern China. This strain has been successfully cultured and sequenced [112].

In Moscow region, the predominant tick species are *I. ricinus* and *Dermacentor reticulatus*, which carry *B. afzelii* and *B. garinii*. The infection rate is approximately 30% in ticks, with peak activity observed from early spring to mid-autumn, when temperatures range between 20 and 25 °C and relative humidity is 50% or higher [113].

In the Russian Far East—specifically in Khabarovsk, Vladivostok, and Yuzhno-Sakhalinsk—*B. garinii* and *B. afzelii* have been isolated from *I. persulcatus* ticks and wild rodents [65,66].

*Borrelia miyamotoi*, the agent of HTBRF, has been identified in *I. persulcatus* using molecular methods [114].

#### 2.2.2. Afghanistan

Currently, there are no documented cases of LB in Afghanistan. However, STBRF, caused by *B. microti* and transmitted by *O. erraticus*, has been reported [115].

#### 2.2.3. Armenia

The first cases of LB were documented in 2010, based on clinical presentation and serological confirmation [67]. STBRF has been described in the country, caused by *B. caucasica* and transmitted by *O. verrucosus* [96,116].

#### 2.2.4. Azerbaijan

In eastern Azerbaijan and northwestern Iran, *B. caucasica*, transmitted by *O. verrucosus*, has been identified. Additionally, *Meriones persicus* rodents have been recognized as reservoirs of a *B. duttonii*-like strain, confirmed through Multilocus sequence analysis [97].

#### 2.2.5. Cambodia (South Hemisphere)

In Cambodia, reports on *Borrelia* infections are limited. A case of tick-borne relapsing fever has been documented [117]. Moreover, a tourist developed LB caused by *B. valaisiana* after being bitten by an *I. persulcatus* tick during a trip to Cambodia [68].

Cases of louse-borne relapsing fever have also been reported in the country [116].

#### 2.2.6. China

China is home to more than 100 tick species. The most representative species in the continental region include *I. granulatus*, *Dermacentor marginatus*, *Dermacentor silvarum*, and *Haemaphysalis longicornis*. Environmental factors influencing tick presence and distribution include urbanization, agricultural land-use, forest cover, annual temperature range, and precipitation. Under climate warming scenarios, tick distributions have shifted northward, while deforestation has contributed to population declines in central and southern China [118].

*Borrelia* species identified in China include members of the LG, the RFG, and species outside these classifications. The first documented case of LB occurred in 1986 in Heilongjiang Province, northeastern China, in a patient presenting with erythema migrans following a tick bite. Since then, hundreds of EM cases have been reported in the region. Molecular analyses have confirmed the presence of *B. garinii*, *B. afzelii*, and *B. valaisiana*, with at least one case demonstrating human pathogenicity [119].

A novel LG species, *B. sinica*, was identified through molecular methods in ticks and rodents. This species was cultured from *I. granulatus* and rodents such as *Apodemus agrarius* and *Niviventer confucianus*, collected along the Yangtze River valley. Transmission electron microscopy revealed four periplasmic flagella inserted at each end of the spirochete [120,121]. Genetic and amino acid diversity in the p66 gene have been observed among Chinese strains of *B. garinii* and *B. afzelii* [122]. In northeastern provinces such as Jilin and Heilongjiang, *I. persulcatus* ticks have tested positive for *Borrelia* DNA. Phylogenetic analysis confirmed the presence of *B. garinii*, *B. afzelii*, *B. bavariensis*, and *B. bissettii* [69].

*Borreliae* of the RFG, including both hard tick-borne relapsing fever and soft tick-borne relapsing fever, have also been identified. HTBRF includes *B. miyamotoi* (vector: *I. persulcatus*; reservoir: *Apodemus agrarius*), which is pathogenic to humans [123]. *B. theileri*, pathogenic to livestock, has been found in *Rhipicephalus* (*Boophilus*) *microplus* and *Apodemus agrarius*.

*B. persica*, a member of the STBRF group (vector: *Ornithodoros tholozani*), is pathogenic to humans and dogs [98] and has been detected in wild small mammals such as *Rhombomys opimus* and *Meriones libycus* [98].

In Hubei province, Candidatus *B. fainii*, a novel species pathogenic to humans, was identified in two bats. This species belongs to the RFG and shows sequence similarity to New World STBRF species [40,124].

Unclassified *Borrelia* species have been detected in *Haemophysalis concinna* and *Niviventer confucianus*. The wide distribution and diversity of RFG *Borrelia* in China pose a significant public health concern [98].

A new species, Candidatus *B. javanense*, has been isolated from *Amblyomma javanense* ticks infesting *Manis javanica* pangolins. Phylogenetic analysis indicates that this species does not belong to either the LG or RFG, suggesting a distinct lineage. Its pathogenicity to humans remains unknown [125].

#### 2.2.7. Cyprus

Relapsing fever was first diagnosed in Cyprus in 1939, primarily in rural areas around Kyrenia, Famagusta, Nicosia, and Larnaca. Several cases were reported among British soldiers in 1945. The vector, *O. tholozani*, inhabits caves, holes, and rodent burrows. The causative agent has not been definitively identified, with *B. persica* and *B. hispanica* both considered potential pathogens [101].

#### 2.2.8. India

In Arunachal Pradesh, ticks collected from mithun (*Bos frontalis*) and the Tibetan yak (*B. grunniens*), include *I. acutitarsus*, *I. ricinus* and *Rhipicephalus* (*Boophilus*) *microplus*, *Rhipicephalus* (*Boophilus*) *geigy*, *Rhipicephalus sanguineus*, *Haemaphysalis davisi*, *Haemaphysalis darjeeling*, *Haemaphysalis longicornis*, and *Haemaphysalis bispinosa* [126]. Evidence of LB in Arunachal Pradesh includes seroprevalence of anti-Bbsl antibodies and the presence of *I. ricinus*, the primary vector of LB in Europe [70]. LB cases have been indeed reported in northern India, frequently involving the nervous system. Articular and cardiac involvement occurred in 27% and 16% of cases, respectively [127]. Diagnosis was based on ELISA and Western Blot detection of anti-Bbsl antibodies in serum and cerebrospinal fluid [128].

Patients with EM and anti-*Borrelia* antibodies have been observed in Haryana [129]. LB is also known to occur in southern India, particularly in the forested areas of Nagarahole and Bandipur, near Rajiv Gandhi National Park [71].

STBRF has been documented in Kashmir, primarily caused by *B. persica*, transmitted by *O. tholozani*, which inhabits caves, ruins, and rodents dens [100].

#### 2.2.9. Indonesia

On Sulawesi island, several hard tick species have been collected, including *Rhipicephalus* (*Boophilus*) *microplus*, a livestock parasite and vector of *Borrelia theileri*, and *I. granulatus*, a vector of LG spirochetes [20,72]. In the Bogor district, a novel *Borrelia* species was detected in *Amblyomma varanense*, which parasitizes the lizard *Varanus salvator*, common in urban areas of Indonesia and other Asian countries. Phylogenetic analysis revealed a monophyletic group within the REPG. This study highlights the potential public health risks posed by reptile-associated ticks, especially given the increasing global trade in reptiles as pets [130].

#### 2.2.10. Iran

On the Iranian Caspian Sea coast, several hard tick species, including *I. ricinus*, have been found feeding on various mammals, such as sheep, goats, cattle, camels, horses, dogs, donkeys, rodents, and small mammals. PCR analyses have identified *B. bavariensis*, *B. garinii*, *B. afzelii*, *B. valaisiana* (agents of LB), and *B. miyamotoi* (HTBRF agent) [73].

*B. microti*, transmitted by *O. erraticus* is endemic in East Azerbaijan province in Iran and causes STBRF [97]. In northwestern Iran, *B. persica* and *B. balthazardi*, transmitted by *O. tholozani*, have been identified in rodents such as *Meriones persicus* and other small mammals. *B. latyschewii*, transmitted by *O. tartakovsky*, has also been reported [97].

#### 2.2.11. Iraq

Serological evidence has documented LB in humans [131], while molecular methods have detected *Borrelia* infection in stray dogs in Nineveh Province [74]. The geographic distribution of *O. tholozani* closely mirrors the occurrence of clinical relapsing fever caused by *B. persica*. This tick species is extensively distributed across Iraq [100].

#### 2.2.12. Israel

Isolated cases of LB have been reported in humans [75] and in dogs [76], although further studies are needed to confirm these findings. *B. persica* has been isolated in culture from humans and in ticks. Its infection [99] transmitted by *O. tholozani* has been documented in humans [100,102], as well as in dogs and cats [132]. Rock hyrax (*Procavia capensis*) has been identified as a reservoir host for *B. persica* [133].

#### 2.2.13. Japan

The first case of Lyme disease in Japan dates back to 1987. Isolations from erythema migrans lesions [77] and from *I. persulcatus* confirmed the presence of *B. garinii* and *B. afzelii*. Japanese strains of *B. garinii* differ immunologically and genetically from European strains in their outer surface protein A, while *B. afzelii* isolates from Japan and Europe are identical. *B. japonica*, a novel LG species, with unknown human pathogenicity, was isolated from *I. ovatus* [134].

In eastern Hokkaido, *B. lonestari*-like organisms have been detected in wild deer (*Cervus nippon yesoensis*) [135] and in *Haemaphysalis flava* and *Haemaphysalis megaspinosa* ticks [136].

A novel HTBRF agent, *B. miyamotoi* sp. nov. (strain HT31T) was identified in *I. persulcatus* [137]. Retrospective serological surveillance revealed high anti-GlpQ seroprevalence in the Chubu region, where *B. miyamotoi* was found in *I. persulcatus* ticks, marking the first such report in Japan [138].

Imported cases of relapsing fever due to *B. persica* have been described in Japan, although no native cases have been documented [139].

*Borrelia* spp. have also been reported in reptiles and *Amblyomma* ticks, which are suspected vectors of REP-group *Borreliae* [140].

#### 2.2.14. Jordan

In southeastern Jordan, *I. ricinus* ticks and anti-Bbsl antibodies in humans have been documented [78]. *Borrelia turcica* (REPG) has also been found in blood and organs of *Testudo graeca* exported from Jordan [36].

*B. persica*, the agent of the STBRF, transmitted by *O. tholozani*, has also been identified. Diagnosis is primarily based on blood smear microscopy [100].

#### 2.2.15. Kazakhstan

The prevalence of *Borrelia* spp. in Ixodid ticks collected from southeastern Kazakhstan (Almaty Oblast) was studied using conventional PCR targeting the 16S rRNA gene to differentiate Bbsl genospecies. LB agents were detected exclusively in *I. persulcatus*, while Bbss was not found in any tick pools. Partial sequencing of the 16S rRNA gene revealed the presence of *B. miyamotoi*, *B. afzelii*, and *B. garinii*, with *B. afzelii* being the dominant genospecies in the Zailiyskiy Alatau, Dzungarian Alatau, and Yenbekshikazakh districts [79].

Almaty Oblast is considered endemic for LB, transmitted by *I. persulcatus* in Almaty city and the Talgar and Karasay districts [141]. In the Zhambyl region, a case of meningoencephalitis with co-infection by *B. burgdorferi* and tick-borne encephalitis virus has been reported [142].

#### 2.2.16. Korea

LB is endemic in the northeastern alpine region of Korea. Clinical manifestations include EM in 55% of patients and neurological symptoms in 36%. A case of acrodermatitis chronica atrophicans has also been reported [80].

*B. burgdorferi* sensu lato has been isolated from *I. persulcatus*, and EM cases with anti-*Borrelia* antibodies have been documented [143]. LB is particularly prevalent among forest workers in national parks [144].

In the Chungbuk and Kangwon provinces, *B. afzelii* and *B. garinii* have been identified and characterized in *Ixodes* ticks and in *Apodemus agrarius* mice [81]. *B. garinii*, *B. bissettii* (LG), and *B. miyamotoi* (RFG) have also been detected in *I. nipponensis* ticks [145].

The raccoon dog (*Nyctereutes procyonoides*), a wild canid native of East Asia and Europe, is a potential reservoir for zoonotic pathogens. *Haemaphysalis flava* and *Haemaphysalis longicornis* ticks collected from raccoon dogs have tested positive for *B. theileri* and other pathogens [146]. A study conducted at the United States Army Garrison Humphreys (Republic of Korea), used molecular and genotypic analyses to detect *Borrelia* spp. in hard ticks collected from Korean water deer (*Hydropotes inermis argyropus*) between January 2018 and December 2019. *B. afzelii* and *B. miyamotoi* were identified in *I. nipponensis* [147,148].

#### 2.2.17. Kuwait

In 1996, a 25-year-old woman who tended sheep developed relapsing fever caused by *B. duttoni*. However, reports of STBRF in the Arabian Gulf region remain rare [103].

#### 2.2.18. Kyrgyzstan

In Kyrgyzstan, borreliosis is represented by STBRF, caused by *B. microti*, transmitted by *O. erraticus* [104].

#### 2.2.19. Laos (Southern Hemisphere)

Cases of LB have been documented in Laos, primarily caused by *B. afzelii* [63]. In the Nakai District, Khammouan province, a *B. lonestari*-like spirochete (RFG) was isolated from *Amblyomma* spp. and *Haemaphysalis* spp. ticks collected from the wild deer, *Cervus nippon yesoensis* [63].

#### 2.2.20. Malaysia (Southern Hemisphere)

Two patients with LB and erythema migrans have been reported, and *B. afzelii* has been identified in blood donors [149]. A serological survey for anti-*Borrelia burgdorferi* antibodies in Orang Asli, the indigenous people of Peninsular Malaysia, indicated a positivity rate of 8% (73 out of 904 volunteers), although human LB cases in rural Malaysian communities remain poorly documented [82].

In *I. granulatus* ticks collected from the forests of Gunung Gading National Park and Kubah (Sarawak), and from small mammals (especially in *Rattus tanezumi*) on oil palm plantations in Sarawak (Malaysian Borneo), *Borrelia yangtzensis* (an LB agent) was detected. *B. miyamotoi* (HTBRF agent) was found in *Sundamys muelleri* [150,151] and identified in *Haemaphysalis hystricis* ticks, collected from wild boars in the Orang Asli community [59,152].

#### 2.2.21. Mongolia

The first LB cases were detected in 2007 in the Zavkhan and Selenge Provinces through serological testing for anti-Bbsl antibodies [83]. In 2009, *B. bavarensis* (was identified in ticks and rodents [84].

The primary vector is *I. persulcatus*. Molecular analyses of ticks collected in the Selenge and Bulgan Provinces revealed the presence of *B. afzelii*, *B. bavariensis*, and *B. garinii* (LG), as well as *B. miyamotoi*, belonging to the Siberian strain of the HTBRF [153,154]. *B. miyamotoi* has also been detected in *Haemaphysalis longicornis* and *Dermacentor nuttalli* ticks using molecular methods applied to both humans and tick samples [155].

#### 2.2.22. Nepal

The first documented case of LB was reported in 2018 in a 32-year-old woman from Pokhara, Kaski District. The patient recalled a lesion consistent with erythema migrans on her left thigh, but did not remember a tick bite. ELISA and Western Blot tests for anti-Bbsl antibodies, along with PCR for *Borrelia* detection, were positive [85].

#### 2.2.23. New Guinea

Reports have suggested the presence of anti-Bbsl antibodies in HIV-positive patients, although often cases were not confirmed by Western Blot. These may represent false positives, and the presence of borreliosis in New Guinea requires further confirmation [156].

#### 2.2.24. Oman

The first case of LB (Lyme arthritis) in Oman was described in 2022 in a 10-year-old girl [86].

#### 2.2.25. Pakistan

In Khyber Pakhtunkhwa, *B. theileri* was identified in hard ticks (*Rhipicephalus microplus* and *R Rhipicephalus turanicus*) collected from domestic animals [20]. In the same province, *Amblyomma gervaisi* ticks, collected from *Varanus bengalensis* lizards, tested positive for *Borreliae* of the REPG, as confirmed by PCR and phylogenetic analysis [157].

*Argas persicus* (vector: *O. tholozani*) infests domestic chickens and ducks, while *Carios vespertilionis* infests bats. These ticks were collected in districts including Peshawar, Mardan, Swabi, Charsadda, Chitral, Lakki Marwat, Bannu, Bajaur, and Hangu. Molecular characterization confirmed the presence of *B. anserina* DNA [38].

A case of louse-borne relapsing fever was reported in Karachi in 1990 [158].

#### 2.2.26. Philippines

Equine tick-borne infections were identified using molecular methods, including the detection of *B. burgdorferi* sensu lato [159].

#### 2.2.27. Saudi Arabia

A case of LB has been reported in a 30-year-old man from Dammam, Eastern Province, presenting with erythema migrans, paresthesia, myalgia, and vertigo [87,88].

Due to the economic importance of camels, tick infestations are closely monitored. *B. theileri*, an HTBRF agent, has been identified in *Hyalomma dromedarii* and *Hyalomma marginatum* ticks, collected in Riyadh [160].

Human cases of STRF caused by *B. persica* have occurred in Saudi Arabia, although their incidence is likely underestimated [105]. In 2014, a case of LBRF due to *B. recurrentis* was reported [161].

#### 2.2.28. Singapore

The presence of LB is rare or uncertain. Seventy-two patients with erythema anulare, resembling EM, tested negative for anti-Bbsl antibodies. In 2012, a patient with neuroretinitis in the left eye tested positive for Bbsl IgG by Western Blot [63].

#### 2.2.29. Syria

No cases of LB have been reported in Syria. However, STBRF caused by *B. persica*, transmitted by *O. tholozani*, has been documented [100].

#### 2.2.30. Tajikistan

A French tourist who visited Tajikistan was diagnosed with *B. persica* infection upon returning to Paris. The diagnosis was confirmed through molecular methods, including sequencing. *B. persica* is typically transmitted by *O. tholozani*, a tick species that inhabits caves, soil, wall crevices, homes, and cow stables [106].

Similarly, an Italian tourist was diagnosed with *B. microti* infection, an agent of tick-borne relapsing fever, following travel to Kyrgyzstan and Tajikistan. The diagnosis was confirmed upon her return by blood smear microscopy and PCR-based methods [104].

#### 2.2.31. Thailand (Southern Hemisphere)

LB has been reported in dogs from Chiang Mai, as confirmed by ELISA and PCR [89]. In the Phop Phra District, Tak Province, *B. miyamotoi* was identified in rodents and *I. granulatus* ticks collected from *Mus caroli*, *Berylmys bowersi*, *Bandicota indica*, and *Leopoldamys sabanus*, which inhabit cultivated areas, increasing the risk of human exposure. Anti-*B. miyamotoi* antibodies were also detected in both humans and rodents [162].

In Lophuri Province, *Borrelia* spp. were detected in *Amblyomma varanense* and *Amblyomma helvolum* ticks collected from aquatic lizards (*Varanus salvator*, *Varanus bengalensis*) and reptiles (*Python reticulatus* and *Python bivittatus*) and *Amblyomma geoemydae* ticks collected from a turtle (*Indotestudo elongata*) [63]. Molecular analyses identified *Borreliae* of the REPG [163].

#### 2.2.32. Türkiye

LB was first described in Istanbul in 2010, confirmed by serological testing and isolation in Barbour–Stonner–Kelly medium from cerebrospinal fluid and skin samples [90]. Between 2009 and 2013, ten cases of LB with erythema migrans appearing 1–2 weeks after tick bites were reported [91]. A case of LB-related uveitis was also documented [93], which is notable given the high prevalence of uveitis due to Adamantiades–Behçet disease in Turkey [164]. LB cases have also been reported in Erzincan, Northeastern Turkey [92].

*B. persica*, an STBRF agent, is present in Turkey and transmitted by *O. tholozani* [100]. *B. turcica*, belonging to the REPG, is transmitted by *Hyalomma aegyptium* ticks and can infect turtles and birds. However, the only reservoir hosts are turtles of the *Testudo* genus [165].

#### 2.2.33. Turkemnistan

STBRF has been documented in association with *B. persica*, vectored by *O. tholozani*, and *B. latyschewii*, transmitted by *Ornithodoros tartakovskyi* [100], which is known to inhabit rodent burrows.

#### 2.2.34. Uzbekistan

In Gava, located in the foothills of Namangan Province, cases of *B. persica* infection have been reported. The vector is *O. papillipes*, a tick with anthropophilic behavior [107].

## 3. Discussion

Vector-borne pathogens maintain transmission cycles between arthropod vector and vertebrate reservoir hosts. The movement of host and vector contributes to the geographical expansion of infectious agents and the emergence of new diseases. In Eurasia, bacteria such as *Borrelia* spp. infect a wide range of vertebrates, including birds, rodents, and larger mammals, including humans, highlighting the critical role of host–vector associations in the northward and southward expansion of tick habitats and the spread of diseases, such as borreliosis [166].

Environmental and climatic factors, including temperature, humidity, latitude, and altitude significantly influence the tick survival and distribution, thereby reshaping their ecological niches. Additionally, increased travel involving infected animals from endemic regions, the introduction of new vectors, and anthropogenic environmental changes have contributed to the emergence and proliferation of canine vector-borne diseases, posing a growing risk to human health [167]. Ecological niche models (ENMs) can be applied, with this regard, to predict the geographic distribution of tick species and understanding the environmental factors that influence their presence and the transmission of tick-borne diseases and the transmitted pathogens [168].

Europeans are increasingly exposed not only to the direct effects of climate change, but also to its indirect consequences, such as the spread of climate-sensitive infectious diseases with epidemic potential [169]. Studies have confirmed the presence of borreliosis across Europe, Northwest Asia, and Southeast Asia, although its prevalence in Southeast Asia appears lower. This may be attributed to limited research and underreporting in countries such as Vietnam, the Philippines, Cambodia, Myanmar, Brunei, and East Timor [63].

In Eurasia, *Borrelia* spp. have been identified in hard ticks species, including *I. granulatus*, *I. persulcatus*, *I. ricinus*, *Haemaphysalis* spp., *Amblyomma varanense*, *A. testudinarium*, *Dermacentor auratus*, and *Rhipicephalus* spp. Members of the relapsing fever group have also been found in soft ticks of the families *Asgasidae* (*Ornithodoros* spp., *Argas* spp.) and the newly described family *Nuttalliellidae* [170].

Within the Lyme group, three major genospecies—*B. afzelii*, *B. bavariensis*, and *B. garinii*—likely of Asian origin, utilize distinct tick vectors in Asia (*I. persulcatus*, *I. granulatus*, *I. acutitarsus*) and Europe (*I. ricinus*). These ticks require high relative humidity (≥80%) and are typically found in forested environments [44]. The bacteria infect a wide range of vertebrates and cause Lyme borreliosis, the most prevalent vector-borne disease in the Northern Hemisphere. The geographic expansion of LB agents is facilitated by host–vector associations, reinforcing their role in the emergence and spread of vector-borne pathogens [166].

LB is present in all European countries, and has been reported in 24 Asian countries, although case numbers in some regions may be underestimated [63]. In Europe, *I. ricinus* is the primary vector, transmitting both Bbsl and Bbss genospecies, whereas *I. persulcatus*, prevalent in Asia does not transmit Bbss.

In Central Asian and Middle East, tick-borne relapsing fever is primarily caused by *B. persica*, although other species such as *B. microti*, *B. latyschewii*, *B. baltazardi*, and *B. caucasica* have also been described. The taxonomy of *Borrelia* spp. is complex and based on the co-speciation. Species-specific PCR targeting the *glpQ* gene is commonly used to differentiate *B. persica* from *B. microti*, the two most prevalent species in Asia [171].

The distribution of vectors and reservoirs of the three *Borrelia* groups—Lyme group, relapsing Fever group, and Echidna–Reptile Group—varies between the Northern and Southern Hemispheres and near the equator. This review focuses on their distribution in Eurasia. LB is associated with hard ticks (*Ixodes* spp.), which prefer humid environments but exhibit ecological plasticity, allowing for adaptation to suboptimal conditions. These ticks are widespread in the Northern Hemisphere and decline in prevalence toward the equator and Southern Hemisphere. In contrast, relapsing fever is more common in tropical and arid regions, favored by *Ornithodoros* spp. ticks. Notably, while LB has been reported in four Southern Hemisphere Asian countries (Cambodia, Laos, Malaysia, Thailand), STBRF has not been documented in these regions. Climate change has facilitated the emergence and geographic expansion of vector-borne diseases, including LB and other borrelioses, by altering the distribution of tick vectors [169]. RFG *Borrelia* spp. can be transmitted by soft ticks (STBRF), hard ticks (HTBRF), or lice (LBRF). STBRF is typically transmitted by *Ornithodoros* spp., which prefer dry climates [172]. HTBRF agents such as *B. theileri* and *B. miyamotoi* share vectors with LB agents. While *B. theileri* infects livestock, *B. miyamotoi* is pathogenic to both humans and animals. STBRF is also present in Central Asia and the Middle East [171].

The REPG has been documented in Indonesia, Japan, Pakistan, Thailand, Turkey, and China. Most LBRF cases are imported, particularly from African countries such as Somalia and Eritrea. In Asia, LBRF was reported during the Second World War, with sporadic cases currently documented in countries like Saudi Arabia and Pakistan [173].

*B. recurrentis* is not transmitted by ticks; the human body louse is its only confirmed vector, and humans are the sole known reservoir [174]. *B. javanense*, isolated from *Amblyomma javanense* ticks, does not belong to either the LG or RFG, and its pathogenicity to humans remains unknown [125].

All major *Borrelia* groups are present in both Europe and Asia. LB is highly endemic across Europe and is reported in 24 Asian countries. STBRF is present in five European and 20 Asian countries. *B. turcica*, belonging to the REPG, has been identified in Greece and Turkey, infecting tortoises (*Testudo* spp.), and is transmitted by *Hyalomma aegyptium* ticks [175].

## 4. Conclusions

The distribution of borreliosis across Eurasia provides a valuable model for studying the spread and evolution of tick-borne diseases in the context of climate change. The presence and prevalence of different *Borrelia* species are closely linked to climatic conditions that influence the life cycles and habitats of their respective vectors. Understanding these ecological and epidemiological dynamics is essential for anticipating future disease emergence and implementing effective public health strategies.

## Figures and Tables

**Table 1:** Hard and soft ticks and transmitted *Borreliae* to humans in Europe and Asia.

Hard Ticks	Geographical Area	Borreliae Infecting Humans	Group	Reservoirs Animals
*I. ricinus*	Western Europe	*B. afzelii*, *Bbss*, *B. garinii*, *B. lusitaniae*, *B. spielmani*, *B. bavarensis*, *B. valaisiana.*	LG	*Apodemus flavicollis*, *Apodemus sylvaticus*, *Clethrionomys glareolus*, *Turdus merula*
	Iran, India, Jordan, Turkey	*B. bavariensis*, *B. garinii*, *B. afzelii*, *B. valaisiana*	LG	Rodents, dogs
	Western Europe, Iran	*B. miyamotoi*	HTBRF	Rodents, birds, deers
*I. persulcatus*	Eastern Europe, Russia	*B. garinii* (European strain 20047) *B. afzelii*, *B bavariensis*	LG	*Tamias sibericus*,
	Cambodia, China, Japan, Korea, Kazakhstan	*B. garinii* (Asian strain NT 29), *B. afzelii*	LG	*Apodemus agrarius*, *A. speciosus*, *Niviventer confucianus*, *Turdus merula*
		*B. miyamotoi*	HTBRF	Rodents, birds
*I. frontalis*	Germany, Switzerland, Portugal	*Bbsl*, *B. lusitaniae*	LG	Passerin birds, *Turdus merula*, *T. philomelos*, *Parus major* and *Fringilla coelebs*
*I. pavlovsky*	Russia	*B. garinii*	LG	
*I. uriae* [22]	Faroe Island	*B. garinii*	LG	*Fratercula arctica*
*I. granulatus* [23]	China, Indonesia, Laos, Malaysia, Nepal, Singapore, Thailand	*B. valaisiana*,	LG	Wild rodents (*Niviventer confucianus*), migratory birds, *Varanidae* (Lizard)
*I. ovatus*	China, India, Japan	*B. miyamotoi*	HTBRF	Dogs, cats, rodents and birds [24]
*I. columnae* [25]	Japan, China, Korea, Thailand	*B. valaisania* (Strain Am 501)	LG	Rodents: *Suncus murinus*, *Mus calori*, *Rattus norvegicus*
*I. nipponensis* [26]	Japan, Korea	*B. afzelii*, *B. garinii*, *B. valaisiana*	LG	*Apodemus agrarius*
**Soft Ticks**				
*O. verrucosus*	Ukraine, Russia	*B. caucasica*	STBRF	Rodents, birds
*O. erraticus*	Iberian Peninsula, Portugal [27]	*B. hispanica*	STBRF	Pigpens, pig farming
	Iran [28], Afghanistan	*B. microti*	STBRF	Pigeons, cattle, sheep, goats, chickens
*O. tholozani* [29,30]	Iran	*B. baltazardii*	STBRF	Rodents
	Middle East, Central Asia India, Israel	*B. persica*	STBRF	Wildlife animals Cairo spiny mouse
*O. tartakovskyi*	Iran, Middle East Pakistan China [31]	*B. latyshevi* [32]	STBRF	Great Gerbil (*Rhombomys opimus*)

**Table 2:** Hard and soft ticks and transmitted *Borreliae* to animals in Europe and Asia.

Hard Ticks	Geographical Area	Borreliae Infecting Animals	Group	Reservoirs Animals
*I. frontalis*	Germany, Switzerland, Portugal	*B. turdi* [33]	LG	Passerin birds, *Turdus merula*, *T. philomelos*, *Parus major* and *Fringilla coelebs*
*I. ovatus*	Japan	*B. japonica*	LG	Mice [34]
*I. tanuki* [35]	Japan	*B. tanuki*	LG	*Apodemus speciosus*
*I. turdus*	Japan	*B. turdi*	LG	Passerin birds, *Turdus merula*, *T. philomelos*, *Parus major* and *Fringilla coelebs*
*Amblyomma gervaisi*	Pakistan	*Borrelia* spp.	REPG	*Varanus bengalensis*, lizards
*Hyalomma aegiptium*	Greece, Turkey, Romania [36]	*B. turcica*	REPG	Turtles (*Testudo græca*), birds
*R. turanicus* [20]	Pakistan	*B. theileri*	HTBRF	Goats
*R. microplus*	Pakistan	*B. theileri*	HTBRF	Cows and sheep
*R. annulatus* [37]	Iran	*B. theileri*	HTBRF	Cattle
*R.* (*Boophilus*) *microplus*	China	*B. theileri*	HTBRF	*Apodemus agrarius*, wild mammals
*Haemaphysalis flava*	Korea	*B. theileri*	HTBRF	Raccoon dogs (*Nyctereutes procyonoides*)
*Haemaphysalis longicornis*	China	*B. theileri*	HTBRF	Wild mammals
**Soft Ticks**				
*O. verrucosus*	Ukraine, Russia	*B. armenica*	Mouse infection	Mice, Guinea pigs, birds
*Argas reflexus*	Worldwide	*B. anserina*	Avian RF	Birds
*A. persicus* [38,39]	Pakistan, China	*B. anserina*	Avian RF	Ducks, domestic fowls
*Carios* (*Alectorobius*) *kelleyi* [40]	China	*B. johnsonii*	Bats RF	Bats
*Carios vespertilionis* [41]	Pakistan, China, UK, France, Sweden	*Borrelia* sp. CPB1	Bats RF	Bats (*Pipistrellus pygmaeus*)

**Table 3:** Lyme group Borrelia distribution in Asian countries.

Geographic Area		Human		Animals/Reservoir	Ticks	Refs.
		Pos ^1^	EM ^2^			
Asian Russia	*B. afzelii*, *B. garinii*, *B. bavariensis*	Yes	Yes	Wild Rodents *Clethrionomys rufocanus*, *Apodemus peninsulae*	*I. persulcatus*, *I. pavlovskyi*, *Dermacentor nuttalli*, *D. reticulatus*	[64,65,66]
Armenia	*B. burgdorferi* sl	Yes	Yes		*Ixodes* spp.	[67]
Cambodia	*B. valaisiana*	Yes		*Niviventer confucianus*,	*I. persulcatus*	[68]
China	*B. garinii*, *B. valaisiana*, *B. bavariensis*, *B. bissettii*, *B. sinica*	Yes	Yes	*Apodemus agrarius*, *A. speciosus*, *Niviventer confucianus*, *Turdus merula*	*I. persulcatus*, *I. granulatus*, *I. columnae*, *I. ovatus*	[69]
India	*B. burgdorferi* sl	Yes	Yes	Rodents (Squirrels, Chipmunks)	*I. acutitarsus*, *I. kashmericus*, *I. ovatus*, *I. ricinus*	[70,71]
Indonesia	*B. burgdorferi sl*	Yes	Yes	Varanid Lizard	*I. granulatus*	[20,72]
Iran	*B. bavariensis*, *B. garinii*, *B. afzelii*, *B. valaisiana*	Yes		Dogs	*I. ricinus*	[73]
Iraq	*B. burgdorferi* sl	Yes		Dogs		[74]
Israel	*B. burgdorferi* sl			Dogs		[75,76]
Japan	*B. afzelii*, *B. garinii*, *B. valaisiana*, *B. tanuki*, *B. turdae*, *B. japonica*	Yes	Yes	*Apodemus speciosus*, *Apodemus ainu*	*I. persulcatus*, *I. nipponensis*, *I. ovatus*, *I. tanuki*, *I. turdus*, *I. columnae*	[77]
Jordan	*B. burgdorferi* sl	Yes			*I. ricinus*	[78]
Kazakhstan	*B. afzelii*, *B. garinii*,	Yes	Yes		*I. persulcatus*	[79]
Korea	*B. afzelii*, *B. garinii*, *B. a valaisiana*, *B. bissettii*	Yes	Yes	Rodents (*Apodemus agrarius*), Migratory Birds, Dogs	*I. nipponensis*, *I. persulcatus*	[80,81]
Laos	*B. afzelii B. yangtenesis*	Yes		Wild Rodents (*Niviventer confucianus*), Migratory Birds *Lizard Reptiles*	*I. granulatus*	[63]
Malaysia	*B. yangtzenesis*, *B. afzelii*	Yes	Yes	Rodents (*Rattus tanezumi*), Small mammals	*I. granulatus*	[82]
Mongolia	*B. afzelii*, *B. garinii*, *B. bavariensis*	Yes	Yes	Rodents	*I. persulcatus*	[83,84]
Nepal	*B. burgdorferi* sensu lato	Yes	Yes			[85]
Oman	*B. burgdorferi* sensu lato	Yes				[86]
Saudi Arabia	*B. burgdorferi* sl	Yes	Yes		*-*	[87,88]
Singapore	*B. burgdorferi* sl	Yes	Yes		*I. granulatus*, *Haemaphysalis* spp.	[63]
Thailand	*B. burgdorferi* sensu lato			Dogs	*I. granulatus*	[89]
Turkey	*B. burgdorferi* sensu lato	Yes	Yes		*I. ricinus*	[90,91,92,93]

^1^ Positivity; ^2^ Erythema migrans.

**Table 4:** Soft tick-borne relapsing fever in Asian countries.

Countries	Borreliæ STBRF	Ornithodoros Ticks	Humans	Animals/ Reservoirs	Refs.
Asian Russia	*Borrelia latyshewii*	*O. tartakowskyi*	Yes	Wild Rodents *Clethrionomys rufocanus*, *Apodemus peninsulae*	[54,55]
Afghanistan	*B. microti*	*O. erraticus*	Yes	Rodents	[95]
Armenia	*B. caucasica*	*O. verrucosus*	Yes	Rodents	[96]
Azerbaijan	*B. caucasica* *B. duttoni*-like	*O. verrucosus*	Yes	Rodents (*Meriones persicus*)	[97]
China	*B. persica* *B. fainii*	*O. tholozani*	Yes	*Apodemus agrarius*, *A.speciosus*, *Niviventer confucianus*, *Turdus merula* Bats	[98]
Congo	*B. duttoni*	*O. moubata*	Yes	Dogs	[99]
Cyprus	*B. hispanica* *B. persica*	*O. erraticus* *O. tholozani*	Yes	Rodents, dogs, foxes, bats	[100,101]
India	*B persica*	*O. tholozani*	Yes	Porcupines (*Hystrix indica*), rock hyraxes (*Procavia capensis*), rodents (squirrels, chipmunks)	[100]
Iran	*B. persica* *B. baltazardii* *B. latyskewii* *B. microti*	*O. tholozani* *O. tartakowskyi* *O. erraticus*	Yes	Rodents (*Meriones persicus*), dogs	[97]
Iraq	*B. persica*	*O. tholozani*	Yes	Camels, cattle, dogs	[100]
Israel	*B. persica*	*O. tholozani*	Yes	Dogs Jackals (*Canis aureus*), red foxes (*Vulpes vulpes*), rock hyrax (*Procavia capensis*), cats	[100,102]
Jordan	*B. persica*	*O. tholozani*	Yes	Camels, dogs, cattle, *Pipistrellus kuhli*,	[100]
Kuwait	*B. duttoni*	*Ortnithodoros* spp.	Yes	Sheep, rodents	[103]
Kyrgyzstan	*B. microti*	*O. erraticus*	Yes	Rodents	[104]
Pakistan	*B. persica*	*O. tholozani*	Yes	Guinea pigs	[38]
Saudi Arabia	*B. persica*	*O. tholozani*	Yes	Camels, goats	[105]
Syria	*B. persica*	*O. tholozani*	Yes	Dogs	[100]
Tajikistan	*B. persica*	*O. tholozani*	Yes	Dogs, cats	[106]
Turkey	*B. crocidurae* *B. persica*	*O. tholozani*	Yes	Dogs, horses, rodents, turtles	[100]
Turkmenistan	*B. persica* *B. latyschewii*	*O. tholozani* *O. tartakovskyi*	Yes Yes	Dogs, cats, Rodents	[100]
Uzbekistan	*B. persica*	*O. papillipes*	Yes	Camels, cats, cattle, dogs	[107]

## Data Availability

No new data were created or analyzed in this study.

## Abbreviations

The following abbreviations are used in this manuscript:

EM	Erythema migrans
LB	Lyme borreliosis
LG	Lyme Group
RFG	Relapsing Fever Group
REPG	Echidna-Reptile Group
Bbss	*Borrelia burgdorferi* sensu stricto
Bbsl	*Borrelia burgdorferi* sensu lato
STBRF	Soft Tick-Borne Relapsing Fever
HTBRF	Hard Tick-Borne Relapsing Fever
LTBRF	Louse-Borne Relapsing Fever
TBP	Tick-borne pathogens
PCR	Polymerase chain reaction
